# Reactivity of Antibodies Immobilized on Gold Nanoparticles: Fluorescence Quenching Study

**DOI:** 10.3390/molecules31010183

**Published:** 2026-01-04

**Authors:** Dmitriy V. Sotnikov, Andrey S. Agapov, Anatoly V. Zherdev, Boris B. Dzantiev

**Affiliations:** A.N. Bach Institute of Biochemistry, Research Center of Biotechnology, Russian Academy of Sciences, Leninsky Prospect 33, Moscow 119071, Russiazherdev@inbi.ras.ru (A.V.Z.); boris.dzantiev@mail.ru (B.B.D.)

**Keywords:** gold nanoparticles, conjugates, immunoglobulins, fluorescein, fluorescence

## Abstract

Antibodies labeled with gold nanoparticles are widely used in analytical systems. Consequently, the choice of methods for producing such conjugates requires a precise determination of the retained reactivity of the antibodies. Existing methods give highly variable results, necessitating new, simple, and accurate approaches. This study demonstrates how a fluorescein (FL) and anti-FL antibody pair can be used to comparatively evaluate conjugation conditions. The method is based on the quenching of fluorescein emission after binding to antibodies, enabling real-time monitoring of interactions in the reaction medium. Using this approach, we compared a series of conjugates of gold nanoparticles (average diameter 24 nm) and anti-FL antibodies that were obtained with antibody concentrations of 5, 10, and 20 μg/mL during the conjugation. The proportion of antibodies that retained their reactivity varied from 11% to 58%. We also estimated the blocking of the conjugates’ surfaces by two widely used reagents, bovine serum albumin and mercapto polyethylene glycol. It was found that the former provides better retention of antigen-binding activity of immobilized antibodies. The difference between these two kinds of preparations is most pronounced—reaching up to a twofold change—at lower antibody densities.

## 1. Introduction

Antibody-nanoparticle conjugates have become one of the most widely used reagents in bioanalytics [1,2]. Their practical significance has engendered a critical need for quantitative data about their composition and functional activity, enabling the rational production of reagents with pre-defined characteristics, moving away from reliance on empirical recommendations [3,4]. It is known that adsorption-based conjugation, a widely employed method, causes a substantial loss of antigen-binding capacity for the majority (from 60% to 94%) of conjugated antibody molecules [5,6,7,8]. This inactivation phenomenon was found to be associated with a complex interplay of factors, encompassing the proximity of antigen-binding sites to the nanoparticle surface, steric hindrance from neighboring molecules, electrostatic repulsion caused by charged nanoparticles, and partial denaturation of immunoglobulins during the adsorption process [3,5,6,7]. Targeted conjugation employing immunoglobulin-binding reagents can significantly increase the percentage of active antibodies but does not provide a guaranteed improvement, since these reagents themselves are susceptible to activity loss upon immobilization.

To characterize nanoparticle conjugates with biomolecules, diverse analytical techniques have been proposed, including absorption and emission spectroscopy, surface-enhanced Raman spectroscopy, dynamic light scattering, and a spectrum of analytical separation methods (such as chromatography, electrophoresis, ultracentrifugation, field-flow fractionation), mass spectrometry, immunoanalytical methods, and other approaches—see reviews [9,10,11]. Despite the large amount of data obtained by these techniques, significant discrepancies often arise between the results obtained from different methods, leaving many questions unresolved [12,13].

Fluorescence spectroscopy, lauded for its exceptional sensitivity and minimal background interference [14], is one of the most attractive methods in the investigation of nanoconjugate properties. We have earlier proposed its applications to characterize the composition and activity of nanoparticle-antibody conjugates. Specifically, a method based on measuring the fluorescence of tryptophan residues has been developed for analyzing conjugates of antibodies against protein antigens [7]. However, the interactions of such conjugates with high-molecular antigens can be hampered by steric constraints of the binding. Therefore, the binding capacity of conjugated antibodies against low-molecular antigens requires special study. As an efficient antigen for this purpose, we chose an intensely fluorescent substance, fluorescein [8]. The method proposed for its study was based on the same sequence of actions: incubation of the antibody conjugate with the antigen, separation of the formed complexes via centrifugation, and subsequent quantification of residual—in this case, fluorescein—fluorescence in the supernatant. A disadvantage of these methods is the likelihood of partial dissociation of the immune complex during the centrifugation step, which introduces additional variability into the obtained results.

For a clear answer to the question about the degree of antibody inactivation during conjugation, a method is needed that allows differentiation between bound and free antigen in solution without separating components of the reaction medium. Such a possibility can be provided by the registration of fluorophore quenching in immune complexes. This effect was found for antibodies to fluorescein, which reduce its fluorescence by more than an order of magnitude [15]. This means that the fluorescence of a mixture of fluorescein and antibodies against it is almost completely determined by the concentration of free fluorescein. The applicability of this approach to determine the activity of anti-fluorescein antibodies and their conjugate with gold nanoparticles was confirmed in [16], but only as a proof-of-principle demonstration for one preparation. The presented study is aimed at a multivariate analysis of anti-fluorescein antibodies labeled with gold nanoparticles under different antibody: gold nanoparticle ratios and for the subsequent treatment with bovine serum albumin or mercapto polyethylene glycol, which are the most commonly used stabilizers of gold nanoparticle conjugates [17,18,19].

## 2. Results and Discussion

### 2.1. Characterization of GNPs

**Figure 1:**
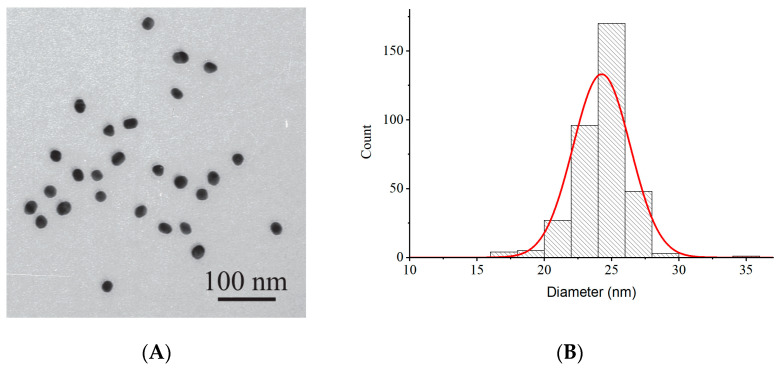
Characterization of GNPs by transmission electron microscopy. (**A**) Micrograph. (**B**) Particle size distribution histogram. The red curve is an approximation by a Gaussian distribution.

Gold nanoparticles were synthesized by the citrate reduction method and then characterized by transmission electron microscopy (TEM). The synthesis in our study involved boiling for 25 min, followed by cooling to room temperature over about half an hour. According to literature data [20], 20 min is sufficient for complete reduction of the gold salt with citrate in boiling water under the conditions of the Frens method. The pH of the nanoparticle solution after synthesis was 5.4. Before conjugation, the pH was adjusted to 9 with potassium carbonate to avoid aggregation, since GNPs are more stable in an alkaline environment due to the negative charge on their surface. The TEM data indicated an average diameter of GNPs (calculated from measurements for 355 nanoparticles) equal to 24 nm (Figure 1). The standard deviation did not exceed 2 nm, confirming the homogeneity of the resulting carriers for antibody immobilization.

The average hydrodynamic diameter of the resulting nanoparticles, determined by dynamic light scattering (see Figure S1 (Supplementary Materials)), was 28.2 nm. The size distribution had no additional peaks, further indicating the monodispersity of the preparation and the absence of nanoparticle aggregation.

### 2.2. Testing the Influence of Antigen–Antibody Interaction on Fluorophore Fluorescence

Since the fluorescence of a molecule is sensitive to its environment, changes in optical properties of a fluorophore are often used to detect intramolecular rearrangements and intermolecular interactions [21,22,23]. The changes caused by incorporation of the fluorophore into specific complexes allow monitoring these interactions in real-time mode directly in solution without separation of reagents.

**Figure 2:**
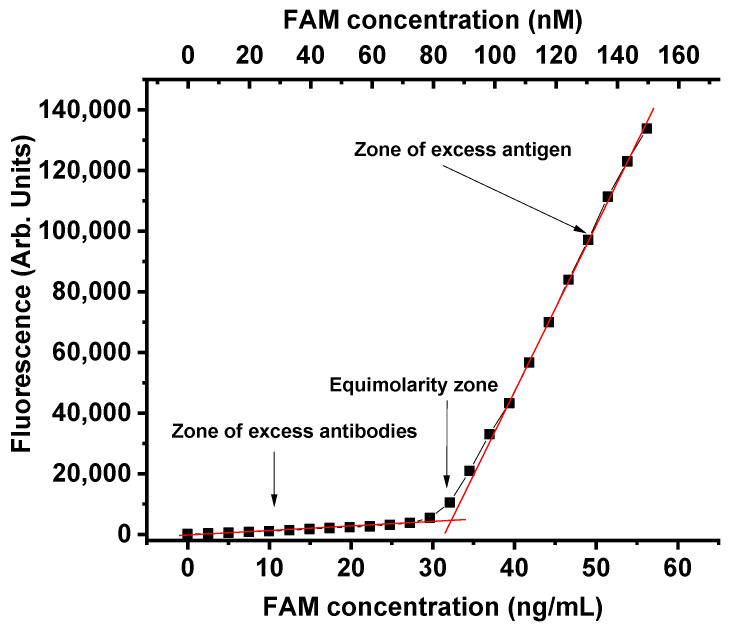
Fluorescence of solutions containing different concentrations of added FAM and 10 μg/mL anti-FAM antibodies. Red lines are linearizations of areas of excess antibodies and excess antigen.

The characterization of the fluorescein–anti-fluorescein pair was started by titrating the antibodies through the successive addition of aliquots of 5-carboxyfluorescein (FAM), which is a molecular form of fluorescein. Figure 2 displays three clearly distinguishable ranges on the titration curve, representing zones of excess anti-FAM antibodies, approximately equal concentrations of immunoreagents, and excess antigen (FAM). The fluorescence signal in the first and third zones depended linearly on the FAM concentration. In contrast, near the equimolar point, a deviation from both linear dependencies was observed.

The complete emission spectra of free and bound fluorescein, as well as the concentration dependence of fluorescein fluorescence, showing high agreement with the linear approximation, were presented in our previous article [16]. The observed linearity of the concentration dependence over the studied concentration range provides clear evidence that a correct interpretation of the results of the changes presented in the article does not require consideration of additional factors arising in concentrated fluorophore solutions. The error in fluorescence measurements over the entire range of concentrations used is less than 0.5%. The detection limit of free fluorescein based on its fluorescence under our experimental conditions is 10 pg/mL.

### 2.3. Technique of Experiments for Determining the Antigen-Binding Capacity of Anti-Fluorescein Antibodies

The total fluorescence of the solution minus the background (F) can be represented as the sum of the fluorescence of free (F_f_) and bound (F_b_) FAM molecules. The fluorescence of free FAM is equal to the product of its concentration (C_f_) and the fluorescence of a single concentration (F_1_). Bound FAM fluoresces 1/X times less intensely than free FAM. Therefore:

F = F_1_ × C_f_ + X × F_1_ × C_b_, (1)

where C_f_ and C_b_ are the concentrations of free and bound FAM, respectively, and X is the coefficient of fluorescence reduction after the transition of FAM from its free form to its bound form.

The ratio of the slopes (tangents) for linearized dependencies in the zones of antibody excess and antigen excess gives the coefficient of fluorescence reduction after FAM binds to antibodies (X). In our investigations (see Figure 2 and the data below), the measured coefficients X were in the range of 0.02–0.04, meaning that binding to free or conjugated antibodies reduced fluorescence by 96–98%. The X values measured in each experiment were used in the following calculations. It is important to note that the fluorescence quenching of fluorescein upon binding to antibodies is not a unique effect of this anti-FL antibody clone. The same processes have been previously described and used to monitor the thermodynamics and kinetics of the binding [24,25]. Fluorescence is highly dependent on the composition of the medium, and any solution components, including dissolved salts, affect the absolute fluorescence value. However, the proposed method for calculating the concentrations of free and bound hapten is based not on absolute fluorescence, but on its relative change during the transition from the bound form of FAM to the free one. These differences are accounted for by the coefficient “X” in Formulas (1) and (2). This coefficient is calculated individually for each solution with its unique composition. Therefore, absolute fluorescence does not affect the calculation results.

Taking into account that C_f_ + C_b_ is the total concentration of FAM initially added to the reaction mixture (C), we can transform Equation (1) to the following formula for C_f_ that uses only known and measurable values:

C_f_ = (F/F_1_ − X × C)/(1 − X). (2)

This formula allows calculating the concentration of free FAM. The difference between this value (C_f_) and the initially added concentration of FAM (C) gives the concentration of bound FAM (C_b_). In the zone of antigen excess, C_b_ is equal to the total concentration of antigen-binding centers, taking into account that one immunoglobulin G molecule contains two such centers.

The value of F_1_ is equal to the slope of the linear part of the antibody titration curve in the zone of antigen excess. This is because the concentration of bound FAM in this zone has already reached its maximum and remains constant at all points.

Note that the proposed approach enables the measurement of the concentration of reactive antigen-binding centers in a single experiment, without the requirement of obtaining a calibration curve beforehand. This feature sets this development apart from other approaches [3,5,12], where calibration curves may differ significantly in solutions of different compositions and so need to be obtained anew each time.

### 2.4. Description and Justification of the Design of Experiments to Test Different GNP-Antibody Conjugates

The GNP-antibody conjugates were synthesized using three concentrations of anti-FAM antibodies: 5, 10, and 20 μg/mL. According to a summary consideration of previous research data conducted in [7], when forming a monolayer, the antibodies (IgG molecules) occupy an area on the surface of about 20–25 nm^2^. For GNPs with a diameter of 24 nm, used at a concentration corresponding to OD = 1.0 at 522 nm and thus contained in the amount of 3.6 × 10^11^ nanoparticles per 1 mL of solution, such an antibody load is achieved at an IgG concentration of about 6.5–8.2 μg/mL. Thus, the selected concentrations are: 5 μg/mL—less than the filled monolayer, 10 μg/mL—slightly more than the filled monolayer, and 20 μg/mL—significantly more than the filled monolayer.

Aggregation of colloidal systems is a major problem limiting the stability of nanoparticle conjugates of biomacromolecules. For this reason, after obtaining conjugates, they are usually treated with high concentrations of blocking compounds to stabilize the suspension and prevent interactions that lead to aggregation. However, additional binding of these blocking components (stabilizers) to conjugates can interfere with the antigen-binding activity of immobilized antibodies and even displace the antibodies from the nanoparticle surface. Therefore, the evaluation of not only the initially synthesized but also the stabilized GNP-antibody conjugates is in demand. In our study, two reagents frequently used as conjugate stabilizers, bovine serum albumin (BSA) and mercaptopolyethyleneglycol (PEG-SH) [26,27,28,29,30,31,32], were considered. After incubating the GNPs with antibodies for 30 min, the conjugates were stabilized by adding 0.25% BSA or 0.25% PEG-SH. Thus, a total of six conjugate variants were obtained, differing in the content of antibodies and the applied stabilizer.

For each conjugate, four samples were analyzed: (i) pure antibodies of the corresponding concentration; (ii) the reaction mixture of GNPs, antibodies, and stabilizer before separation by centrifugation; (iii) the supernatant, and (iv) the GNP-antibody conjugate purified by centrifugation. Analysis of each of the preparations allows us to obtain the following data regarding the process of conjugating antibodies with GNPs:

- the binding capacity of pure antibodies allows estimating the proportion of active antigen-binding sites in the original antibody preparation;
- the binding capacity of the reaction mixture containing both conjugated and free antibodies provides a direct evaluation of the proportion of antibodies that lose activity as a result of their binding to nanoparticles;
- the binding capacity of the supernatant obtained after separation of the conjugate by centrifugation allows estimating the proportion of antibodies unbound to nanoparticles (assuming centrifugation does not affect the binding capacity of antibodies);
- the binding capacity of the purified conjugate reflects the final activity of the conjugated antibodies.

### 2.5. Experimental Characterization of Stabilized GNP-Antibody Conjugates

**Figure 3:**
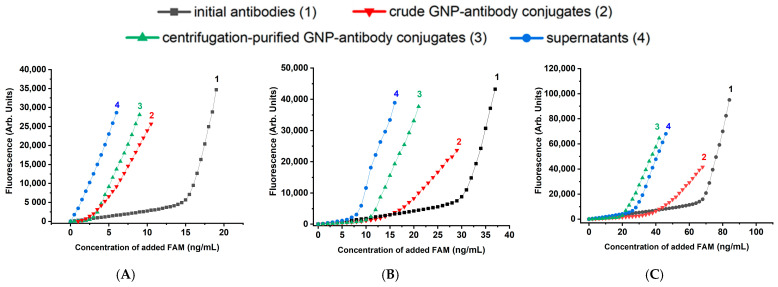
FAM fluorescence in solutions of pure antibodies (1), reaction mixtures after mixing MoAb and GNP (2), purified MoAb-GNP conjugates (3), and supernatants after centrifugation of conjugates (4), obtained at anti-FAM antibody concentrations: 5 μg/mL (**A**), 10 μg/mL (**B**), and 20 μg/mL (**C**). Stabilizer—0.25% BSA.

Each of the four solutions (initial antibodies, unpurified GNP-antibody conjugates, GNP-antibody conjugates purified by centrifugation, and supernatants), was titrated with FAM solution to determine the binding capacity of antibodies in them. The obtained titration curves for the case of BSA stabilization are shown in Figure 3. As can be seen, bends in the curves were observed for all solutions. These bends correspond to the zone of equivalence between the molar concentrations of the antigen and the active antigen-binding sites of the antibodies.

**Figure 4:**
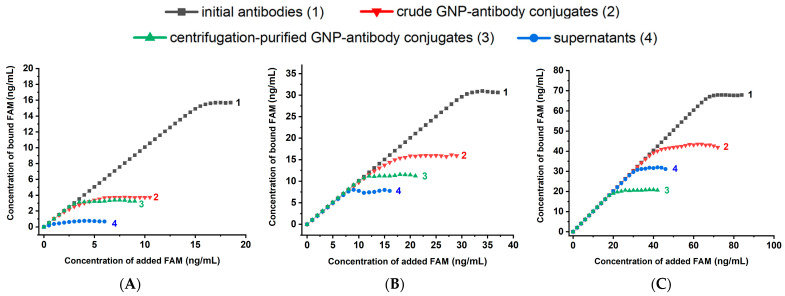
Dependence of the concentration of bound FAM on the initially added concentration for: initial antibodies (1), crude GNP-antibody conjugates (2), centrifugation-purified GNP-antibody conjugates (3), and supernatants (4) at anti-FAM antibody concentrations: 5 μg/mL (**A**), 10 μg/mL (**B**), and 20 μg/mL (**C**). Stabilizer—0.25% BSA.

The concentration dependences of fluorescence were recalculated using Equation (2), and the dependences for the bound FAM concentrations were obtained. As can be seen from Figure 4, all 12 curves demonstrated reaching saturations. The concentration of bound FAM (C_b_), corresponding to the upper plateau on the titration curves, reflects the concentration of available binding sites in a given solution. Quantitative data on the maximum binding capacity of free and GNP-bound antibodies are summarized in Table 1.

**Figure 5:**
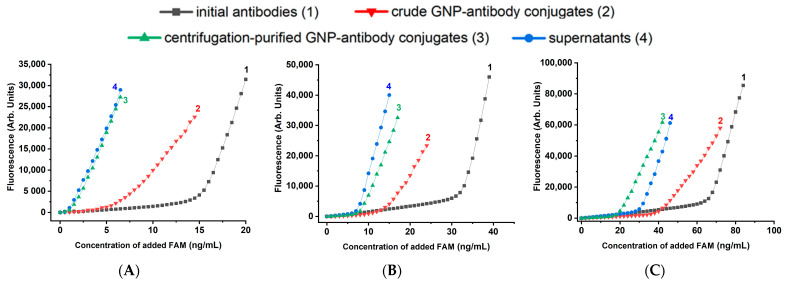
FAM fluorescence in solutions of initial antibodies (1), reaction mixtures after mixing the antibodies and GNPs (2), purified antibody-GNP conjugates (3), and supernatants after centrifugation of the conjugates (4), obtained at anti-FAM antibody concentrations: 5 μg/mL (**A**), 10 μg/mL (**B**), and 20 μg/mL (**C**). Stabilizer—0.25% PEG-SH.

**Figure 6:**
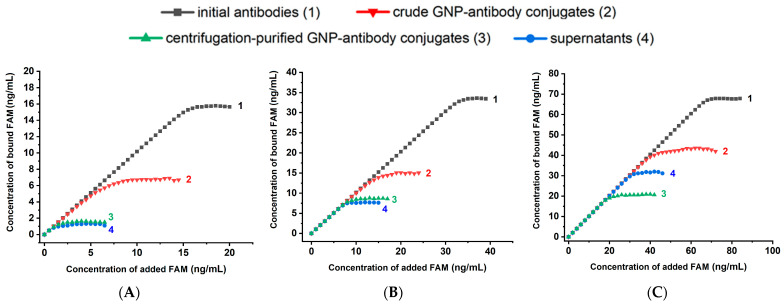
Dependence of the concentration of bound FAM on the initially added one for: initial antibodies (1), crude GNP-antibody conjugates (2), centrifugation-purified GNP-antibody conjugates (3), and supernatants (4) at anti-FAM antibody concentrations: 5 μg/mL (**A**), 10 μg/mL (**B**); and 20 μg/mL (**C**). Stabilizer—0.25% PEG-SH.

The conjugates stabilized with PEG-SH were studied according to the same protocol. The titration curves of the conjugates are shown in Figure 5 and Figure 6, and the amounts of bound FAM in the studied solutions are summarized in Table 2.

The obtained GNP-antibody conjugates with both BSA and PEG-SH were stable, with the exception of one preparation. For the conjugate obtained by adding 5 μg/mL antibodies and 0.25% PEG-SH, significant aggregation was observed. After centrifugation, it was not possible to completely resuspend the sediment into the solution. Therefore, the binding capacity values of the conjugate for this preparation are underestimated.

To confirm the absence of nonspecific interactions of FAM with BSA and PEG-SH adsorbed on the surface of GNPs, an additional experiment was conducted. The fluorescence of GNP-BSA 0.25% and GNP-PEG-SH 0.25% solutions in the absence of antibodies was measured with the addition of various concentrations of FAM. The obtained dependences (see Figure S2 in the Supplementary Materials) show that fluorescence depends linearly on the FAM concentration. This confirms the absence of nonspecific binding of FAM. to BSA and PEG-SH.

Because one of the preparations exhibited partial aggregation (the preparation obtained using 5 μg/mL antibodies during synthesis, stabilized with PEG-SH), additional spectroscopic studies of the conjugates were conducted. The absorption spectra of native GNPs and their conjugates are shown in Figure S3 (Supplementary Materials). All conjugates exhibited a 6–8 nm shift in the absorption peak, which is typical for GNP conjugates with proteins [33]. For most conjugates, the spectral shape was similar to that of native GNPs, confirming the stability of the colloidal preparations. Only the aggregated preparation exhibited a significant drop in optical density and spectrum broadening, further confirming the presence of aggregates in the solution and a significant loss of GNPs due to sedimentation.

Free antibodies in the supernatants have the same binding capacity per unit concentration as the initial antibodies used for the syntheses. Therefore, the ratio of the concentration of the bound hapten in the supernatant to the concentration of the hapten being bound by the initial antibody preparation gives the fraction of free (not GNP-bound) antibodies after the conjugation. Accordingly, the remaining fraction of initially added antibodies is bound to the nanoparticles.

The determined proportions of antibodies remaining in the supernatant after conjugation and centrifugation are presented in Table 3. Comparing these values allows for evaluating the efficiency of different conjugation variants. As can be seen from the table, at an antibody concentration of 5 μg/mL, virtually all antibodies are immobilized on GNPs. At a concentration of 20 μg/mL, more antibodies are sorbed than can be accommodated in a monolayer (based on the antibody size and the surface area of the nanoparticles). This indicates the possibility of forming multiple layers during antibody sorption under these conditions.

Note that recalculation of molar concentrations shows that even for free antibodies, there is no equality between the concentration of bound antigen and the concentration of added antigen-binding sites. Since we used monoclonal antibodies, which are identical molecules with the same affinity, the discrepancy between the molar concentrations of the added antigen-binding centers and the maximally bound antigen can be explained by a partial loss of antibody activity in our preparation. This may be due to a partial loss of the immunoglobulin structure during storage [34,35]. Thus, a 5 μg/mL (33.3 nM) antibody solution, taking into account the bivalency of IgG, contains about 67 nM of antigen-binding centers but binds a maximum of about 16 ng/mL FAM (42.5 nM). That is, about 37% of the valences of antibodies in the original preparation are inactive. The method considered in this study is one of the few methods that allow determining the true binding capacity of antibodies.

One of the unexpected results is that the sum of bound FAM for free and conjugated antibodies is somewhat greater than that for the initial reaction mixture (see Table 1 and Table 2, last column). That is, the total number of antigen-binding sites in the sediment and supernatant increased compared to non-centrifuged conjugates. This effect was observed for all preparations except for the aggregated conjugate (5 μg/mL of antibodies and PEG-SH as a stabilizer). The difference was more pronounced for solutions with a higher concentration of antibodies. Only the conjugate with 5 μg/mL of antibodies and BSA as a stabilizer demonstrated approximate equality of the compared values. It is noteworthy that in all experiments the titration curve of non-centrifuged conjugates is flatter, and the non-linear region of the curve is wider than for other solutions. This indicates a lower constant of immune interaction for unpurified conjugates. This effect requires more detailed study. One possible explanation is that the effect is related to the formation of protein layers around the nanoparticles, known as the “hard corona“ and “soft corona” [36,37,38,39]. The first portions of antibodies bind tightly to the nanoparticle surface (“hard corona”); then, after covering most of the surface, some of the antibodies form an outer layer of weakly bound molecules (“soft corona”). The outer layer makes the molecules of the first layer less accessible to interact with the antigen, but as a result of harsh physical action (centrifugation), the weakly bound antibody molecules are desorbed and end up in solution, making the molecules of the inner layer more accessible. Other possible explanations for the observed phenomena may be the restructuring of the protein coat during centrifugation or changes in the protein globule itself, since mechanical influences can change the structure of immunoglobulins [40].

In general, it can be concluded that the conjugates with BSA are more stable than the conjugates with PEG-SH. They show less aggregation and a higher binding capacity.

### 2.6. Assessment of the Degree of Reactivity Retention for Antibodies Conjugated with GNPs

The concentration of FAM bound in the supernatant (C_bs_) is proportional to the concentration of free antibodies after conjugation with nanoparticles. If we denote the concentration of FAM bound to the initial unconjugated antibodies as C_bi_, then the ratio C_bs_/C_bi_ gives the proportion of free antibodies after conjugation. Accordingly, the value (1 − C_bs_/C_bi_) is the proportion of antibodies bound to nanoparticles.

The proportion of conjugated antibodies that retained activity after binding to nanoparticles can be calculated in two ways. The first approach is a direct measurement of the concentration of bound FAM in a solution of purified conjugate. This method allows one to estimate the activity of the conjugate after centrifugation:

(3) $$\begin{array}{c} Activity\;of\;conjugated\;antibodies\;after\;centrifugation\\ \;\;\;\;\;\;\;=\frac{Concentration\;of\;FAM\;bound\;by\;conjugated\;antibodies\;(with\;concentration\;\left[C\right])}{Concentration\;of\;FAM\;bound\;by\;free\;antibodies\;(with\;concentration\;\left[C\right])} \end{array}$$

The second approach is based on the fact that the binding capacity of the reaction mixture before separation of free and conjugated antibodies is proportional to the total concentration of active antibodies (free and bound ones). Accordingly, the difference in concentrations of added antibodies and active antibodies in the reaction mixture gives the concentration of antibodies that have lost activity as a result of conjugation. (The expression in parentheses in Formula (4)). Subtracting this value from the total concentration of conjugated antibodies gives the concentration of active conjugated antibodies before centrifugation.

(4) $$\begin{array}{c} Activity\;of\;conjugated\;antibodies\;before\;centrifugation\\ \;\;\;\;\;\;\;=Total\;concentration\;of\;conjugated\;antibodies\\ \;\;\;\;\;\;\;-\;(Concentration\;of\;FAM\;bound\;by\;free\;added\;antibodies\\ \;\;\;\;\;\;\;-Concentration\;of\;FAM\;bound\;by\;crude\;conjugate) \end{array}$$

Thus, the method for estimation of antibody activity proposed in this work also allows for evaluating the effect of centrifugation. The results of this evaluation for conjugates obtained with the use of two stabilizers are presented in Table 4.

According to the obtained data, the binding of antibodies to GNPs leads to inactivation of 61% to 79% of antigen-binding centers. These results are consistent with other studies confirming the loss of activity of most antibodies upon their adsorption conjugation with GNPs [5,8,12]. However, centrifugation of the conjugates leads to a significant decrease in the proportion of inactive valences in the absence of colloid aggregation. Presumably, this is due to the rearrangement of the protein corona around the nanoparticles. It is noticeable that an increase in the concentration of added antibodies leads to an increase in the proportion of active centers for purified conjugates—see the rightmost column of Table 4. Thus, the density of antibody molecules on the GNP surface is also an important factor determining the reactivity of the conjugates.

Aggregation of one of the preparations (conjugate with 5 μg/mL antibodies and PEG-SH as a stabilizer) resulted in a loss of activity by more than threefold as compared to the non-centrifuged conjugate. The aggregation was caused specifically by the centrifugation, since in the non-centrifuged conjugate preparation, about 39% of the antibody valences retained their activity.

### 2.7. Reproducibility of Measurements for Conjugates’ Composition and Activity

**Figure 7:**
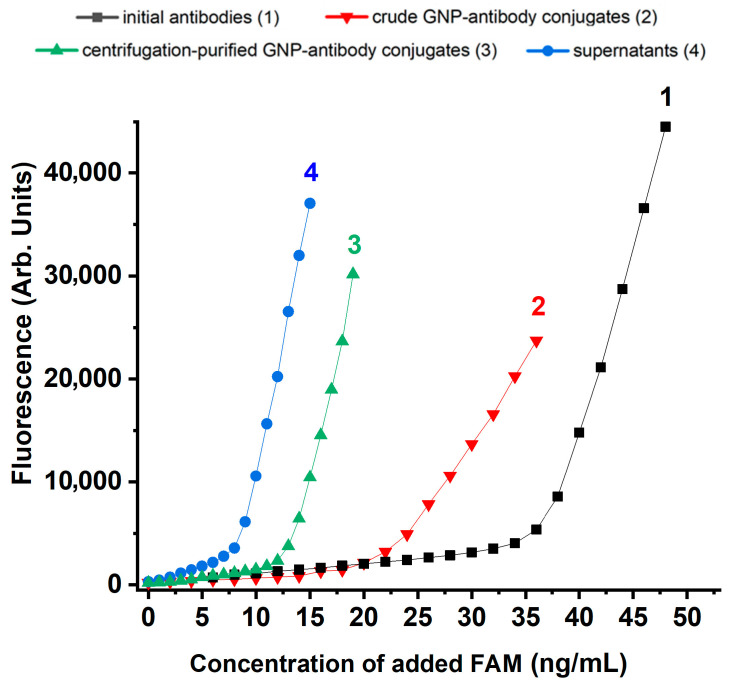
FAM fluorescence in solutions of pure antibodies (1), reaction mixtures after mixing MoAb and GNP (2), purified MoAb-GNP conjugates (3), and supernatants after centrifugation of conjugates (4), obtained with an additional new batch of anti-FAM antibody at a concentration of 10 μg/mL. Stabilizer—0.25% BSA.

To assess reproducibility of the measurements, we additionally tested GNP conjugates with the same antibody clone but from a different batch. The immobilization conditions studied were the following: an antibody concentration of 10 μg/mL, and 0.25% BSA was used as a stabilizing solution. The results obtained for testing the antibodies, their conjugates before and after centrifugation, and supernatants are shown in Figure 7.

The second batch of antibodies demonstrated slightly higher activity than the first. The maximum concentration of bound FAM was 37.3 ng/mL, compared to 33.6 ng/mL for the first batch. Under conditions of maximum FAM binding, its concentration in the supernatant was 9.0 ng/mL; in the uncentrifuged conjugate, it was 22.1 ng/mL; and in the centrifuged conjugate, 13.6 ng/mL. These values indicate that 2.4 μg/mL of antibody remained in the supernatant, while the concentration in the GNP-conjugate was 7.6 μg/mL. Antibody activity in the conjugate before centrifugation was 46%; after centrifugation it increased to 48%. Thus, despite some variability, the repeat experiment showed good agreement with previously obtained results (see Section 2.6).

### 2.8. The Impact of the Proposed Technique on the Knowledge About the Properties of Antibody-GNP Conjugates

Consideration of the available data from different investigations of the composition and binding capacity of antibody conjugates with GNPs demonstrates significant differences in the results obtained by different methods. Lu Zhang et al. [12], studying the sorption of fluorescein-labeled IgG on GNPs with an average diameter of 15 nm, showed that the determined composition of conjugates differs by more than threefold for three methods used: 3.9 IgG per GNP for measuring the fluorescence of the conjugate after dissolving the GNPs with sodium cyanide, 9.9 IgG per GNP for enzyme immunoassay, and 13.3 IgG per GNP for measuring the fluorescence of the supernatant after separating the conjugate by centrifugation. The use of NanoOrange fluorescent dye for detection after dissolving the GNPs in a KI/I_2_ solution and Bradford colorimetry of unbound antibodies after separating the conjugate, showed twofold different quantities—the sorption of 309 and 660 IgG per GNP with an average diameter of 60 nm [13]. In [5], repeated use of Bradford colorimetry showed the sorption of only 227 IgG per GNP of the same diameter.

The presented study uses a direct, simple, and accurate technique for determining the amount and activity of free and conjugated antibodies. The results obtained with its application in this work allow, on the one hand, to recommend this technique for further comparative study of various conjugation methods, and on the other hand, to formulate two identified patterns that are potentially significant for various preparations.

The obtained data demonstrate that antibodies can partially lose antigen-binding activity even before conjugation. Thus, antibody preparations obtained at different times and with different shelf lives can differ significantly in their properties. Previously used techniques for determining the activity of nanoconjugates with antibodies tacitly assume that all antibodies are initially active.

It was also shown that subsequent manipulations with the conjugate (such as centrifugation and stabilization) also change the amount and binding capacity of bound antibodies. The presence in the conjugate of antibody subpopulations with different affinities to the nanoparticle is a factor changing the conjugates’ activity during storage and manipulations with them.

Accurate and unified assessment of the binding capacity of both free and conjugated antibodies alleviates existing limitations in the characterization of antibody–nanoparticle conjugates. In addition, minimizing the number of manipulative steps in the analytical workflow reduces cumulative experimental variations, enhancing the reliability and reproducibility of the results.

## 3. Materials and Methods

### 3.1. Materials

Monoclonal antibodies against fluorescein were from HyTest (Moscow, Russia). Gold (III) chloride (HAuCl_4_), Tris, and sodium citrate were from Sigma-Aldrich (St. Louis, MO, USA). Poly(ethylene glycol) thiol (PEG-SH; MW 5 kDa) was from Creative PEGWorks (Durham, NC, USA), bovine serum albumin (BSA) was from Boval Biosolutions (Cleburne, TX, USA). 5-carboxyfluorescein (FAM) was from Lumiprobe (Moscow, Russia). All additional reactants for the preparation of working solutions were from Khimmed (Moscow, Russia). Deionized water produced by Milli-Q system (Millipore; Burlington, MA, USA) was used to prepare working solutions.

### 3.2. Preparation of Gold Nanoparticles (GNPs)

Gold nanoparticles were synthesized according to the Frens method [41]. A solution of chloroauric acid (0.2 mL, 5%) was added to water (97.5 mL), heated to boiling, and then a sodium citrate solution (1.5 mL, 1%) was added with stirring. The mixture was boiled for 25 min, cooled to room temperature, and stored at 4 °C.

### 3.3. Characterization of GNPs by Transmission Electron Microscopy (TEM)

GNP preparations were applied to nets (300 mesh) covered by a polyvinyl formal film dissolved in chloroform. The images were obtained with a JEM CX-100 transmission electron microscope (Jeol; Tokyo, Japan) at an accelerating voltage of 80 kV and a magnification of 3,300,000. The digital photographs were analyzed using Image Tool 3.0 UTHSCSA software (University of Texas Health Science Center; San Antonio, TX, USA).

### 3.4. Determination of the Hydrodynamic Radius of GNPs by the Dynamic Light Scattering Method

To determine the hydrodynamic diameters (using dynamic light scattering (DLS)) of the nanoparticles, a Zetasizer Nano ZSP (Malvern Instruments, Malvern, UK) nanoparticle analyzer was used. The measurements were conducted at 25 °C, with light scattering recorded at an angle of 173°. The results were processed using Zetasizer software, version 7.11.

### 3.5. Conjugation of GNPs with Anti-Fluorescein Antibodies

The conjugation was implemented based on the protocol from [42] with minor modifications. Firstly, the anti-Fl IgG were dialyzed for 2 h at 4 °C against a 1000-fold volume of 10 mM Tris-HCl buffer, pH 9.0. The solution of synthesized GNPs was diluted with bidistilled water to reach OD = 1.0 at 522 nm. 0.1 M potassium carbonate solution was then added to the resulting GNP solution until pH 9.0 was reached.

The antibody solutions (5 mg/mL; 2, 4 or 8 µL) were added to 2 mL of GNP solution with stirring to a final antibody concentration of 5, 10, or 20 μg/mL. The mixtures were incubated for 30 min at 20–22 °C with stirring. After this, aqueous solutions of PEG-SH or BSA were added to a final concentration of 0.25% [43]. The reaction mixtures were centrifuged for 15 min at 25,000 *g* and 4 °C using Allegra 64R centrifuge (Beckman Coulter, Indianapolis, IN, USA). The supernatant was separated and used to determine the concentration of free (non-bound) antibodies. The remaining sediment (50 μL aliquot) was resuspended to 2 mL, and its binding with FAM was tested as described below.

### 3.6. Testing the Interaction of Free and GNPs-Bound Antibodies with Fluorescein Antigen

One milliliter of tested solution (free antibodies, GNP-IgG conjugate, or supernatant) and 1 μL of fluorescein solution (1–80 μg/mL) were gently added to a quartz cuvette (transverse dimensions 3 × 3 mm) and vigorously mixed. After 30 s of incubation (RT), fluorescence was measured with excitation at 501 nm and emission at 522 nm using a Shimadzu spectrofluorophotometer RF-6000 (Shimadzu Corp., Kyoto, Japan).

## 4. Conclusions

The implemented control of fluorescein quenching by specific antibodies allows for convenient and precise direct monitoring of immune complexes formation without the need for labeling reactants. These advantages make fluorescence quenching an excellent tool for accurately determining antibody activity. Consequently, this technique was successfully applied to characterize the conjugation of antibodies to nanoparticles—an area of significant importance yet remaining poorly understood. Through this work, the true values of the decrease in antibody activity after binding to GNPs, stabilization by two types of macromolecules, and purification of conjugates by centrifugation were determined with high accuracy.

Since the technique detects binding solutions without separating the components of the medium, it provides a consistent assessment of the effect of each manipulation on antibody activity. Therefore, its application for characterizing and comparing the existing variety of methods for obtaining conjugates of antibodies with types of nanoparticles of different sizes, shapes, and chemical compositions, including random and oriented adsorption, covalent and bioaffinity immobilization, appears promising and informative. This article primarily examines the fluorescein—specific antibodies pair as a tool for comparative evaluation of different conjugation methods, which, based on its application, can be used to develop the most promising solutions for other immunoreagents.

## Figures and Tables

**Table 1:** Maximum concentrations of bound FAM in solutions of initial antibodies, unpurified GNP-antibody conjugates, GNP-antibody conjugates purified by centrifugation, and supernatants. Stabilizer—0.25% BSA.

Antibody Concentration, μg/mL (nM)	Maximum Concentration of Bound FAM, ng/mL (nM)				
	Initial Antibodies	GNP-Antibody Conjugate Before Centrifugation	Supernatant After Centrifugation	GNP-Antibody After Centrifugation	(Pellet + Supernatant) Sum
5 (33.3)	15.8 (42.0)	3.8 (10.1)	0.6 (1.6)	3.3 (8.8)	3.9 (10.4)
10 (66.7)	31.0 (82.4)	16.1 (42.8)	8.0 (21.3)	11.6 (30.8)	19.6 (52.1)
20 (133.3)	70.2 (186.5)	42.0 (111.6)	27.6 (73.3)	22.5 (59.8)	50.1 (133.1)

**Table 2:** Maximum concentrations of bound FAM in solutions of initial antibodies, unpurified GNP-antibody conjugates, GNP-antibody conjugates purified by centrifugation, and supernatants. Stabilizer—0.25% PEG-SH.

Antibody Concentration, μg/mL (nM)	Maximum Concentration of Bound FAM, ng/mL (nM)				
	Initial Antibodies	GNP-Antibody Conjugate Before Centrifugation	Supernatant After Centrifugation	GNP-Antibody After Centrifugation	(Pellet + Supernatant) Sum
5 (33.3)	15.8 (42.0)	6.9 (18.3) *	1.3 (3.5)	1.6 (4.3) *	2.9 (7.7)
10 (66.7)	33.6 (89.3)	15.1 (40.1)	7.7 (20.5)	8.8 (23.4)	16.5 (43.8)
20 (133.3)	67.9 (180.4)	43.5 (115.6)	32.0 (85.0)	20.9 (55.5)	52.9 (140.6)

* Partial aggregation of the conjugate was observed. The conjugate was not completely resuspended after centrifugation.

**Table 3:** Quantitative assessment of antibody immobilization on GNPs for different methods of conjugate stabilization.

Antibody Concentration, μg/mL	Proportion of Antibodies in the Supernatant (%)		Proportion of Antibodies in the Conjugate (%)	
	Stabilizer—BSA 0.25%	Stabilizer—PEG-SH 0.25%	Stabilizer—BSA 0.25%	Stabilizer—PEG-SH 0.25%
5	3.8	8.2	96.2	91.8
10	25.8	22.9	74.2	77.1
20	39.3	47.1	60.7	52.9

**Table 4:** Compositions of GNP-antibody conjugates and the degrees of reactivity retention for conjugated antibodies.

Concentration of Added Antibodies, μg/mL	Maximum Concentration of Bound FAM, ng/mL			
	Activity of Antibodies in the Reaction Mixture *, %	Antibodies in the Conjugate, μg/mL	Activity of Conjugated Antibodies Before Centrifugation, %	Activity of Conjugated Antibodies After Centrifugation, %
Stabilizer—BSA				
5	24	4.8	21	22
10	52	7.4	35	50
20	60	12.1	34	53
Stabilizer—PEG-SH				
5	44	4.6	39	11 **
10	45	7.7	29	34
20	64	10.6	32	58

* Activity refers to the degree of retention of binding capacity relative to initial antibodies preparation with the same concentration. ** Partial aggregation of the conjugate was observed.

## Data Availability

The original contributions presented in the study are included in the article; further inquiries can be directed to the corresponding author.

## Supplementary Materials

The following supporting information can be downloaded at: https://www.mdpi.com/article/10.3390/molecules31010183/s1, Figure S1: Distribution of hydrodynamic diameters (DH) of GNPs. Figure S2: Titration of GNP-BSA 0.25% and GNP-PEG-SH 0.25% conjugates with FAM solution. Figure S3: Absorption spectra of GNPs and their conjugates with antibodies stabilized with BSA and PEG-SH.
