# Supplementary material for: Reactivity of Antibodies Immobilized on Gold Nanoparticles: Fluorescence Quenching Study
Source: Molecules. 2026 Jan 4;31(1):183. doi: 10.3390/molecules31010183 (PMC12787391; doi:10.3390/molecules31010183)
Supplement: Supplementary file 1 [file molecules-31-00183-s001.zip › molecules-4005151-supplementary.pdf]

Supplementary material

Reactivity of antibodies immobilized on gold nanoparticles: Fluorescence quenching study

Dmitriy V. Sotnikov*, Andrey S. Agapov, Anatoly V. Zherdev, and Boris B. Dzantiev

A.N. Bach Institute of Biochemistry, Research Center of Biotechnology, Russian Academy of Sciences, Moscow 119071, Russia

* Correspondence: sotnikov-d-i@mail.ru

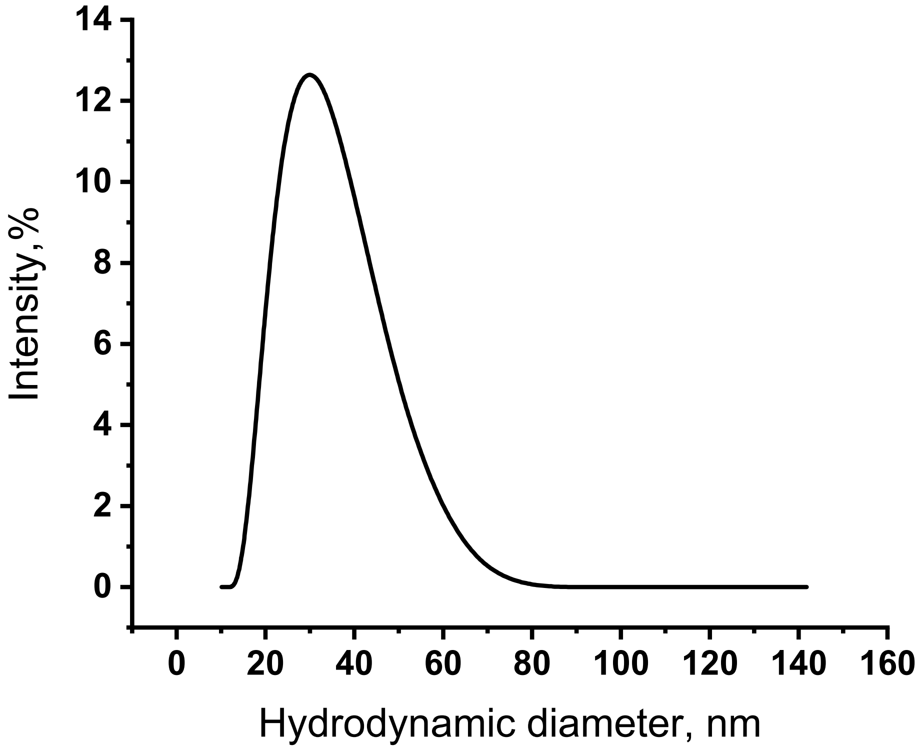

Fig. S1. Distribution of hydrodynamic diameters (DH) of GNPs.
DH = 28.2 ± 10.4 nm, polydispersity (Pd) = 32.4%.

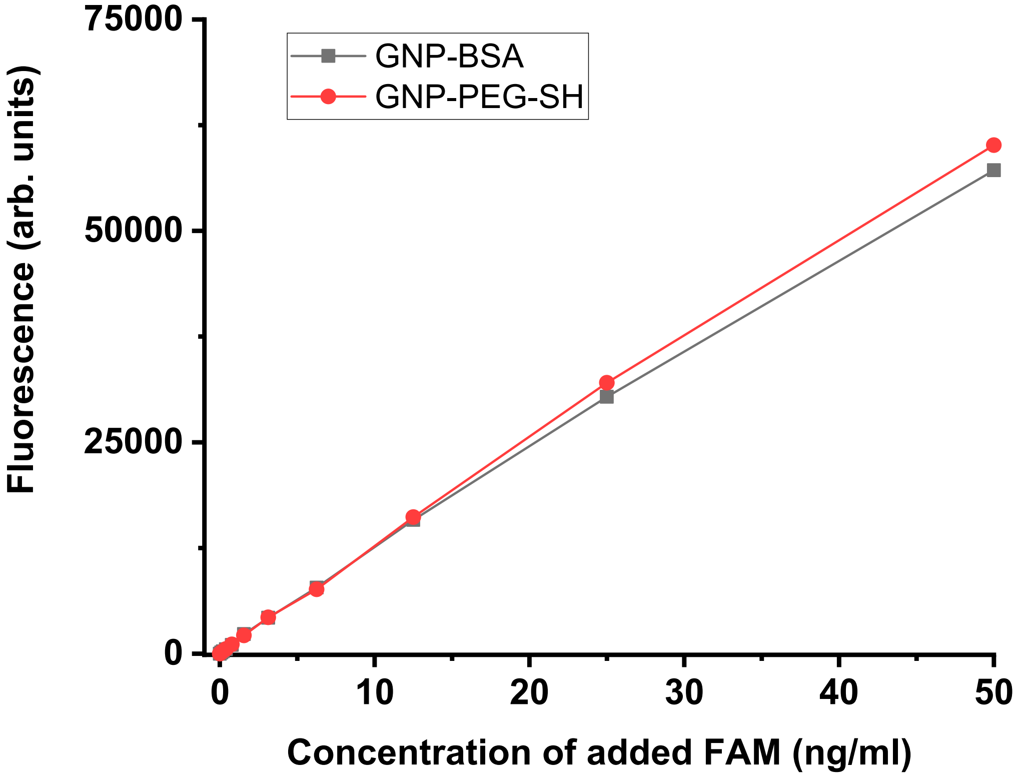

Fig. S2. Titration of GNP-BSA 0.25% and GNP-PEG-SH 0.25% conjugates with FAM solution.

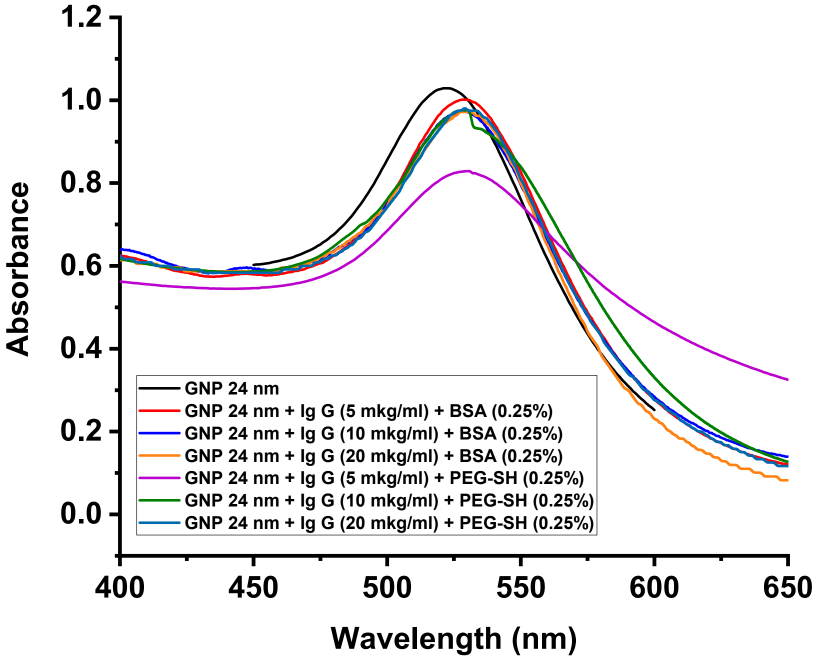

Fig. S3. Absorption spectra of GNPs and their conjugates with antibodies stabilized with BSA and PEG-SH.
